# Depression and anxiety among Pakistani healthcare workers amid COVID-19 pandemic: A qualitative study

**DOI:** 10.1016/j.amsu.2022.103863

**Published:** 2022-05-26

**Authors:** Irfan Ullah, Kiran Shafiq Khan, Iftikhar Ali, Arslan Rahat Ullah, Sonia Mukhtar, Renato de Filippis, Najma Iqbal Malik, Mohammadreza Shalbafan, Zair Hassan, Muhammad Sohaib Asghar

**Affiliations:** a Kabir Medical College, Gandhara University, Peshawar, Pakistan; b Undergraduate Research Organizations, Dhaka, Bangladesh; c Naseer Teaching Hospital, Peshawar, 25000, Pakistan; d Dow University of Health Sciences, Karachi, Pakistan; e Paraplegic Center, Hayatabad, Peshawar, Pakistan; f Department of Medicine, Northwest General Hospital & Research Centre, Peshawar, Pakistan; g University of Management and Technology, Lahore, Pakistan; h Psychiatric Unit, Department of Health Sciences, University Magna Graecia of Catanzaro, Catanzaro, 88100, Italy; i Department of Psychology, University of Sargodha, Sargodha, Pakistan; j Mental Health Research Center, Iran University of Medical Sciences, Tehran, 1449614535, Iran; k Department of Cardiology, Lady Reading Hospital, Peshawar, Pakistan

**Keywords:** Anxiety, Coronavirus, COVID-19, Depression, Health care workers (HCWs)

## Abstract

**Objectives:**

The sudden COVID-19 crisis required a determined effort on the part of the healthcare workers (HCWs) and excessive workload increased the risk of depressive and anxious symptoms in frontliners. The aim of the study was to assess anxiety and depression levels among HCWs during times of pandemic and its potential aggravating factors.

**Materials and methods:**

A web-based survey was conducted to assess the mental health outcomes of healthcare workers and related factors during the COVID-19 pandemic. For assessing depression and anxiety, the Hospital Anxiety and Depression Scale (HADS) comprised of 14 items with seven items for depression and seven for anxiety were used.

**Results:**

Of all 436 participants, 158 (36.2%) showed noticeable symptoms of depression and 220 (50.4%) showed substantial anxiety symptoms. The majority of them were females. It has been observed in the study that female gender, young, and unmarried marital status are associated with higher scores. HCWs working in urban regions show more depressive symptoms. Mild depression and anxiety ratio are very common among participants (21.3%). Factors found to be associated with higher anxiety and depression are the increased number of deceased patients with lower family support.

**Conclusions:**

Altogether, the present study findings present concerns about the psychological well-being of all HCWs during the acute COVID-19 outbreak. Therefore, steps should be taken to protect them from mental exhaustion, so they may fight with more zeal against the infectious pandemic that has caused significant impacts worldwide.

## Highlights

• This study highlighted increasing prevalence of anxiety and depression among frontline COVID-19 healthcare workers.
• Almost one-third had depression and half of them had Anxiety on a validated scale.
• Appropriate measures should be undertaken to protect them from mental exhaustion.

## 1 Introduction

Since December 2019, the world has begun to face a new deadly virus named coronavirus, also known as SARS-2019-CoV, causing Coronavirus Disease (COVID-19) [1]. It first appeared in Wuhan, Hubei Province, China, and then spread worldwide [2]. In January 2020, human-to-human transmission of COVID-19 was officially reported and by that time, a large number of the population had been infected [1] including the medical staff of Wuhan [3]. On March 11, the World Health Organization (WHO) declared this outbreak as a “pandemic” because of the rapid spread of the virus globally [1]. Since then, a large number of infected individuals and associated deaths contributed to enormous emotional stress among the general population [1]. On the other side, the increasing number of patients and pandemic-related deaths, exhausting workload, unavailability of ventilators and intensive care unit (ICU) beds, and shortage of personnel protection equipment (PPE) caused emotional and physical burnout over time among health care workers (HCW) [1,2]. Additionally, the escalating infection rate among medical staff was another factor for such anxiety-related behaviour [4,5].

Previous evidence of severe acute respiratory syndrome (SARS) and the Middle East respiratory syndrome (MERS) outbreak also indicated HCWs to be at higher risk of developing anxiety, depression, burnout, and stress during these periods and these symptoms been differently viewed across gender [[6], [7], [8], [9]]. The initial empirical literature about the COVID-19 pandemic revealed that 53.8% of HCWs rated the psychological impact of the outbreak as moderate to severe [10]. Therefore, upholding the sufficient number of physicians, nurses, clinicians, pharmacists, respiratory therapists, and other clinicians is not only the requirement yet this crisis demands a healthy workforce [11] enabled to work in full potential over an extended period in a rapidly changing practice environment as this differs wholly from what they are familiar with [12]. It is the need of time to train and counsel the healthcare workers for the sake of their mental health on an emergent basis [11].

Therefore, the current study aimed to evaluate the mental health issues of HCWs during the COVID-19 outbreak and to investigate its potential aggravating factors through a web-based cross-sectional research design. Study findings have strong practical and theoretical implications as they will enhance the practical knowledge of policymakers and mental health service providers for efficient dealing of psychological health issues related to HCWs during the COVID-19 outbreak.

## 2 Patients and methods

### 2.1 Study site, participation, and ethics

Researchers adopted a qualitative web-based survey design by using a self-generated online questionnaire. The proportionate convenient sample comprised of HCWs working in hospitals of Pakistan was included in the study and participants were encouraged to share online form links with other colleagues as well. Considering the current situation of the country, face to face community-based survey was not feasible, therefore data were collected using an online Google form within the time frame of 20 April to May 22, 2020 (Pre-COVID-19 vaccination). All the participants that showed up were assessed by using a pre-designed questionnaire and enrolled only after taking informed and e-signed consent. The final sample size was made up of 436 participants. The inclusion criteria in participating were - (i) voluntary willingness to participate, (ii) being an active HCW, and (iii) being a permanent Pakistani resident, and details of sample demographics have been presented in Table 1. The study followed the highest degree of ethical perspective suggested by the Helsinki Declaration, 1975. The research protocol was registered with expedited ethics approval registry of University of Sargodha (UIN: SU/PSY/786-S). Informed consent was obtained, and other ethical issues are highly practiced in the study.

**Table 1:** Comparison of demographic characteristics by Depression and anxiety status (N = 436).

Variables	Depression			Anxiety		
	Absent	Present	*p* value	Absent	Present	*p* value
**Mean Age (S.D)**	4.114 (±2.186)	10.367 (±2.247)		4.042 (±2.355)	8.828 (±0.802)	
**Age**			0.353			0.175
<20	18 (60.0)	12 (40.0)		15 (50.0)	15 (50.0)	
20–24	189 (64.1)	106 (35.9)		151 (51.2)	144 (48.8)	
25–29	52 (59.8)	35 (40.2)		35 (40.2)	52 (59.8)	
≥30	19 (79.2)	05 (20.8)		15 (62.5)	9 (37.5)	
**Gender**			0.055			<0.001
Male	132 (68.8)	60 (31.3)		117 (60.9)	75 (39.1)	
Female	146 (59.8)	98 (40.2)		99 (40.6)	145 (59.4)	
**Marital status**			0.277			0.044
Single	248 (62.9)	146 (37.1)		189 (48.0)	205 (52.0)	
Married	30 (71.4)	12 (28.6)		27 (64.3)	15 (35.7)	
**Resident**			0.219			0.048
Rural	42 (64.6)	23 (35.4)		41 (63.1)	24 (36.9)	
Urban	223 (64.8)	121 (35.2)		164 (47.7)	180 (52.3)	
Semi urban	13 (48.1)	14 (51.9)		11 (40.7)	16 (59.3)	
**Region**			0.385			0.010
Punjab	47 (59.5)	32 (40.5)		48 (60.8)	31 (39.2)	
Sindh	154 (67.2)	75 (32.8)		101 (44.1)	128 (55.9)	
KPK	52 (59.1)	36 (40.9)		51 (58.0)	37 (42.0)	
Islamabad	12 (57.1)	9 (42.9)		7 (33.3)	14 (66.7)	
**Smoking**			0.371			0.700
Non-Smoker	255 (62.8)	151 (37.2)		203 (50.0)	203 (50.0)	
Current Smoker	13 (76.5)	04 (23.5)		08 (47.1)	09 (52.9)	
Former Smoker	10 (76.9)	03 (23.1)		05 (38.5)	08 (61.5)	
**Monthly Income (Rupees)**			0.090			0.452
≤30,000 (≤178.48)	35 (55.6)	28 (44.4)		31 (49.2)	32 (50.8)	
30,001-50000 (178.49–297.47)	42 (55.3)	34 (44.7)		38 (50.0)	38 (50.0)	
50,001-70000 (297.48–416.46)	30 (61.2)	19 (38.8)		29 (59.2)	20 (40.8)	
70,001-100000 (416.47–594.95)	54 (65.1)	29 (34.9)		35 (42.2)	48 (57.8)	
Above 100,000 (>594.95)	117 (70.9)	48 (29.1)		83 (50.3)	82 (49.7)	
**Previous Psychiatry disease**			<0.001			<0.001
Yes	20 (39.2)	31 (60.8)		4 (7.8)	47 (92.2)	
No	258 (67.0)	127 (33.0)		212 (55.1)	173 (44.9)	
**On Psychiatry Medication**			<0.001			0.001
Yes	04 (17.4)	19 (82.6)		4 (17.4)	19 (82.6)	
No	273 (66.4)	138 (33.6)		212 (51.6)	199 (48.4)	
**Self-reporting health**			<0.001			<0.001
Very good	124 (78.5)	34 (21.5)		109 (69.0)	49 (31.0)	
Good	114 (64.0)	64 (36.0)		89 (50.0)	89 (50.0)	
Acceptable	36 (40.4)	53 (59.6)		15 (16.9)	74 (83.1)	
Poor	3 (42.9)	4 (57.1)		3 (42.9)	4 (57.1)	
Very poor	1 (25.0)	3 (75.0)		0 (0.0)	4 (100.0%	

### 2.2 Sample size estimation

The sample size computed by OpenEpi (version 3) was 384, supposing a response distribution of 50%, with a confidence interval (CI) 95% (p < 0.05), Z value of 1.96, and 5% margin of error. A non-response rate of 15% (N = 58) was added to the calculated sample size to decrease any errors in completing the form, resulting in a final sample size of 442. However, 436 were included in the final analysis.

### 2.3 Measures

The questionnaire booklet consisted of three parts i.e., (i) socio-demographic information sheet, (ii) depression subscale, and (iii) anxiety subscale.

### 2.4 Socio-demographics information sheet

This comprised of questions related to the sample's age, gender, residential area, region, smoking, monthly income, previous psychiatric condition, and presently on psychiatric medication and self-reported health status.

### 2.5 Hospital Anxiety and Depression Scale (HADS)

For assessing depression and anxiety HADS scale consisted of 14 items divided into two subscales with seven items for depression and seven for anxiety. Each item is rated on a four-point Likert scale (0–3) with total scores of depression and anxiety subscale ranging from 0 to 21 individually. The depression and anxiety being classified as normal (0–7), mild (8–10), moderate (11–14), and severe (15–21). The Cronbach's alpha of the HADS in the present study was 0.855 for the total scale, 0.690, and 0.850 for the subscales of depression and anxiety respectively.

### 2.6 Statistical analysis

All the collected information was assessed and analyzed via the software Statistical Package for Social Sciences (SPSS, Chicago, Illinois, USA) version 24. Frequencies and proportions of the categorical variable were computed to present the data in a tabulated form. Spearman rank correlation was computed to see the relationship between depression and anxiety with psychiatric medication and the psychological health status of HCWs. Fisher exact tests were used in case each cell having less than 20% expected value. Besides, binary logistic regression was performed to see the Odds ratio and confidence interval of the variables. The level of statistical significance was set at *p* ≤ 0.05.

## 3 Results

A total of 436 healthcare workers participated in the study with the mean age of 23.97 ± 5.875 here ratio of females (55.9%) was higher than males (44%). The majority of the participants were from Sindh (229, 52.52%) followed by KPK and Punjab (20.1% and 18.1% respectively). Table 1 presents the working conditions and environmental effect on healthcare workers' mental health who work in the frontline during times of pandemic. Out of all, 13.8% (n: 13) of the overall sample were former smokers.

The demographic data with depression and anxiety status as shown in Table 1 and among all participants, 158 (36.2%) HCW have symptoms of depression whereas, 220 (50.4%) HCW reported anxiety symptoms.

A subgroup analysis of the pervasiveness of anxiety and depression by age, gender, marital status, residential, and regional groups were further piloted and summarized in Table 1. Where, high prevalence of (145, 59.4%) anxiety and (98, 40.2%) depression among female healthcare workers was observed. The current study found a non-significant mean difference of age, marital status, residence, region and smoking on depression whereas significant mean difference was found on anxiety. Findings revealed that healthcare workers aged between 20 and 24 years old experience more depression (106, 35.9%) and anxiety (144, 48.8%) in contrast to the healthcare workers of age >30 years old who showed less depression and anxiety symptoms (20.8% and 37.5% respectively). Moreover, health care workers who were single/unmarried exhibited more adverse depressive symptoms (146, 37.1%) and anxiety (205, 52.0%) substantiating the significant association (p = 0.044), especially those females HCW's working in the Sindh region followed by Khyber Pakhtunkhwa and Punjab (p = 0.010). Our subgroup analysis further explores that healthcare workers, working in urban regions have a high prevalence of depression (121, 35.2%) and anxiety (180, 52.3%).

Of total HCWs, 21.3% of the participants were mildly depressive, 13.3% reported moderate depression, and 1.6% has severe depressive symptoms. For the anxiety subscale, 21.3% of health care workers were considered to have mild anxiety, 20.2% had moderate anxiety, and 8.9% had severe anxiety symptoms (Table 2).

**Table 2:** Frequency of various depression and anxiety categories (N = 436).

Category	Depression		Anxiety	
	f	%	f	%
Normal	278	63.8	216	49.5
Mild	93	21.3	93	21.3
Moderate	58	13.3	88	20.2
Severe	7	1.6	39	8.9

Table 3 showed the strong positive relationship between HCWs current state of anxiety and depression with their previous psychiatric history and current use of psychiatric medication. This clearly showed that the HCWs with a history of weak psychological or mental health ad on the use of psychiatric medication were more prone to indulge in high depression and anxiety during the COVID-19 pandemic situation.

**Table 3:** Spearman correlation of depression, anxiety, Psychiatry medication and disorder (N = 436).

Variable	1	2	3	4
1. Depression	1			
2. Anxiety	0.573**	1		
3. Previous Psychiatry Disorder	0.229**	0.348**	1	
4. On Psychiatry Medication	0.224**	0.216**	0.552**	1

**p < 0.01.

In Table 4, Logistic Regression analysis illustrates that demographic like being women especially single, having a history of psychiatric disorders were the most independent factors for anxious mental health outcomes.

**Table 4:** Association between demography and anxiety among Health Care Workers.

Variables	*b*	SE	Odds ratios	Odds ratio 95% CI	
				CI Lower	CI Upper
**Gender**					
Male	−0.826	0.197	0.438	0.297	0.644
Female			Reference		
**Marital status**					
Single	0.669	0.337	1.952	1.008	3.783
Married			Reference		
**Resident**					
Rural	−0.910	0.468	0.402	0.161	1.008
Urban	−0.282	0.406	0.755	0.340	1.673
Semi-urban			Reference		
**Region**					
Punjab	−1.130	0.517	0.323	0.117	0.890
Sindh	−0.456	0.482	0.634	0.247	1.629
KPK	−1.014	0.511	0.363	0.133	0.987
Islamabad			Reference		
**Previous Psychiatry Problem**					
Yes	2.667	0.531	14.399	5.087	40.754
No			Reference		
**On Psychiatry Medication**					
Yes	1.621	0.559	5.060	1.692	15.133
No			Reference		

## 4 Discussion

Since the confirmation of the first case of COVID-19 been reported on February 26, 2020, in Pakistan [13]. To combat the deadly illness, many modifications, adjustment, and adaptation processes started in the healthcare systems. Beds and ventilators' capacities have been expanded. Many inpatient zones were labelled as isolation wards to treat COVID-19 patients. Many doctors from medicine specialties were assigned duties in isolation wards with full equipment (PPEs). Despite these things, there is no doubt that this catastrophic and unpredictable condition had an inevitable impression on the mental health of HCWs [11]. The current study's concern about depression and anxiety among health care workers and found that 50.4% of health care workers experienced anxiety and 36.2% experienced depression in the time of pandemic compare to the study conducted in Turkey where 64.7% of the physicians experienced depressive symptoms and 51.6% had anxiety-related symptoms [2]. Another empirical study conducted in China revealed that among 1257 HCWs, 50.4% reported symptoms of depression, and 44.6% reported anxiety [10]. Furthermore, several other studies conducted during previous outbreaks also represent similar findings [7,8,14,15].

Although various cut-off scores and multiple scales have been used for each survey, therefore it is very much possible that massive discrepancy may exist between-studies. Present study findings presented in Table 2, revealed that the majority of the HCWs experienced mild symptoms for both depression and anxiety (21.3%), while moderate and severe symptoms were less common among the participants. This result further emphasized that early detection of such conditions is necessary to avoid any more complex and enduring psychological issues evolving afterwards. As per the current study, the prevalence of mild symptoms of anxiety and depression (21.3%) among HCWs is roughly similar to the corresponding percentages ranging between 22.6% and 36.3% anxiety and 16.5%–48.3% depression by the study reported in China during the same period, which represents the effect of COVID-19 on the general population [16]. Another study piloted in the epicenter, Wuhan, revealed that a majority of HCWs were adversely affected and required mental health support for their psychological turmoils [17].

Furthermore, the current research sub-analysis discovered hypothetically important relations among gender, marital status, residential, and regional differences with depression and anxiety. In the current study, though marital status, residential, and regional differences with depression are non-significant but they reflect mean difference and these findings are in support of previous empirical evidence such as the study of Albert et al. that confirmed the higher prevalence of anxiety and depression in female HCWs [18]. In correspondence, present study results reflect similarities with already established gender gap findings where (145, 59.4%) anxiety and (98, 40.2%) depression appeared to be higher among females. Another study conducted in China by Lai et al. indicated that women and frontline workers are progressing towards unfavorable psychiatric consequences during the COVID-19 outbreak [11]. Furthermore, findings of present research also reflect that single/unmarried HCWs exhibited more adverse depressive symptoms (146, 37.1) and anxiety (205, 52.0) establishing a significant association (*p* = 0.044), especially those females working in the Sindh region followed by Khyber Pakhtunkhwa and Punjab (*p* = 0.010). Previously Elbay et al. also confirmed that being married, having children and HCWs belong to rural areas possess a high level of depression [2]. On the other hand, Liu et al. in their survey, concluded that being single is associated with more odds of depressive symptoms in hospital staff as well [19]. The present study further found that HCWs working in urban regions have a high prevalence of depression (121, 35.2) and anxiety (180, 52.3). On similar lines, several other studies published in a recent month provided data that COVID-19 is rigorously affecting the physical and mental health of doctors, nurses, and other staff members [20,21]. Studies conducted in Hong Kong and Germany also found that medical staff is more vulnerable to anxiety, mental stress, and burnout [[22], [23], [24], [25], [26]], and reported high depressive symptoms and anxiousness during the COVID-19 period. The current study also sought the association of age with depression and anxiety status and found age as one of the potential risk factors for the increased psychological problem during COVID-19. It further found mean difference of age with depression though it was non-significant, yet, this slight mean variation is in support by empirical literature across cultures. For example Huang and Zhao have proclaimed in their study that younger individuals (<35 years) were more likely to acquire anxiety and depression than older ones (≥35 years) [1]. Following the latter study, present study results demonstrate parallel pronouncements that healthcare workers aged 20–24 years experienced high depression (106, 35.9%) and anxiety (144, 48.8%) in contrast to the healthcare workers aged >30 years. Su et al. in their study, also indicated comparable results during the SARS outbreak in Taiwan [27]. Similarly, Elbay et al. in their study, showed concurrence responses that younger HCWs working as frontline force having less professional experience had higher scores of depression [2].

Moreover, healthcare workers living with their spouse displayed lower scale scores than those living alone. Present study findings reported in Table 4, Logistic Regression analysis revealed that being a woman, and single, with a history of psychiatric disorders were the independent analysts for adverse mental health outcomes in our study. These findings are supported by empirical evidence in the literature as Naushad et al. confirmed that healthcare workers especially those working in the intensive care unit and emergency department are likely to develop adverse psychiatric illnesses [28]. Spoorthy et al. in the recent review, also postulated similar findings that HCWs were found to be at higher risk of developing adverse psychiatric outcomes [4]. Studies conducted at the time of the MERS outbreak also shown that healthcare workers of the emergency department were found to have more risk factors of developing posttraumatic stress disorder (PTSD) [29]. Similar incidences were reported during the SARS outbreak when 89% of health care workers were confirmed to be among high-risk individuals who developed psychological ailments [15].

Though current study exploratory findings revealed non-significant mean difference with smoking yet, values of the mean revealed that non-smokers were more progressive to develop high depression (151, 37.2%) and anxiety (203, 50%); healthcare worker having monthly income of above PKR 100000/- showed worse mental health outcomes in all dimensions of interest as they experienced more stress (48, 29.1%) and anxiety (82, 49.7%). Similar findings were also found by previous research that explores the anxiety status across a broad range of HCWs supporting patients with COVID-19 in different global regions [30]. Altogether, the present study findings show concerns about the psychological well-being of all HCWs during the acute COVID-19 outbreak. Furthermore, our subgroup analysis of anxiety and depression based on gender, age, monthly income, history of psychiatric illness, medication history, and severity provided additional valuable insights into particular and potential vulnerabilities of psychiatric and psychological ailments.

## 5 Limitation and future recommendation

Although this study analyzed a large population of HCWs in Pakistan and provides information on the current mental condition of frontline workers during the COVID-19 pandemic, it has several limitations. First, it might have the data from a limited population and the same region/country hence such sample size cannot be based for conclusions. Although using a web-based survey/online system due to the COVID-19 crisis was one of practical feasibility yet it intends to minimize face to face interaction that might result in selection biases. Indeed, the purposive convenience sampling technique does not allow the study population to reflect the actual pattern of the general HCWs population of the country, yet it was the best fit in the current pandemic crisis; however, for a more representative and larger sample, random sampling on large scale is best for generalizability. Second, our study is limited by its cross-sectional nature and its lack of longitudinal follow up. Therefore, it would be ideal to implement a prospective study on the same group of participants after a period. Third, current research used a self-report questionnaire to determine the psychological aspect that did not be dependent on diagnostic criteria by mental health professionals. Fourth, due to the sudden disaster, the research team was ineffectual to assess the psychological health before the outbreak. Fifth that study only evaluates depression and anxiety levels of healthcare workers; thus, further studies are needed that include social support, PTSD and other factors assessment in healthcare workers. Notwithstanding the above limitations, the findings of the current study provide valued information regarding depression and anxiety levels during COVID-19 among HCWs from different specialties across the country.

## 6 Implications

Study findings have strong practical and theoretical implications as they highlighted the high prevalence rates of depression and anxiety among healthcare professionals and raised the special needs advocacy on an emergent basis by taking drastic steps and cutthroat measures for ensuring the mental health of the frontline medical task force [31]. It further will help to enhance the practical knowledge of policymakers and mental health service providers for efficient dealing of psychological health issues related to HCWs during the COVID-19 outbreak and making the work environment more conducive, safe, and stressors free [32]. This will give a cushion to HCWs to be safe from physical exhaustion, mental fatigue, and will be more motivated, energetic to perform duties with excellence [33]. The research team further recommend that healthcare authorities should design multi-disciplinary mental health departments that deal with mental health issues and provide psychological support to healthcare workers. Evaluation should be done via web applications. Regular screening is required to treat and diagnose depression and anxiety in HCWs at the initial level. If diagnosed and addressed timely, it can be treated by psychotherapeutic means.

## 7 Conclusions

In conclusion, the study highlighted the high prevalence rates of depression, anxiety among HCWs and findings showed that females, young, single, and particularly those working in urban areas, were in the risk group and should follow carefully. Furthermore, it was found that the monthly income and smoking status also possess a significant association with depression and anxiety that impart an inevitable emotional impact on HCWs. The most important recommendation would be awareness among HCWs regarding use of personal protective equipment, immunization, and infectivity of the virus. Mental health courses and workshops should be carried out amongst the HCW for helping them to cope up with their mental exhaustion.

## Provenance and peer review

Externally peer reviewed, not commissioned.

## Data availability statement

The data that support the findings of this study are available on request from the corresponding author. The data are not publicly available due to privacy or ethical restrictions.

## Ethical approval

Ethical approval was taken in this study from institutional review board of University of Sargodha (Ref no: SU/PSY/786-S).

## Sources of funding

None.

## Author contribution

I·U, K·S·K, and I.A conceived the idea; A.A, K·S, A.H·P, and S·H collected the data; M.S.A and I·U analyzed and interpreted the data; A.R.U, S.M, R.F, N·I.M, I·U, and M.S did write up of the manuscript; and finally, I·U, Z.H, M.S.A and R.F reviewed and revised the manuscript for intellectual content critically. All authors approved the final version of the manuscript.

## Trail registry number

1. Name of the registry: University of Sargodha
2. Unique Identifying number or registration ID: SU/PSY/786-S
3. Hyperlink to your specific registration (must be publicly accessible and will be checked):

## Guarantor

Muhammad Sohaib Asghar.

## Annals of medicine and surgery

The following information is required for submission. Please note that failure to respond to these questions/statements will mean your submission will be returned. If you have nothing to declare in any of these categories then this should be stated.

## Consent

Studies on patients or volunteers require ethics committee approval and fully informed written consent which should be documented in the paper.

Authors must obtain written and signed consent to publish a case report from the patient (or, where applicable, the patient's guardian or next of kin) prior to submission. We ask Authors to confirm as part of the submission process that such consent has been obtained, and the manuscript must include a statement to this effect in a consent section at the end of the manuscript, as follows: “Written informed consent was obtained from the patient for publication of this case report and accompanying images. A copy of the written consent is available for review by the Editor-in-Chief of this journal on request”.

Patients have a right to privacy. Patients’ and volunteers' names, initials, or hospital numbers should not be used. Images of patients or volunteers should not be used unless the information is essential for scientific purposes and explicit permission has been given as part of the consent. If such consent is made subject to any conditions, **the Editor in Chief** must be made aware of all such conditions.

Even where consent has been given, identifying details should be omitted if they are not essential. If identifying characteristics are altered to protect anonymity, such as in genetic pedigrees, authors should provide assurance that alterations do not distort scientific meaning and editors should so note.

Informed consent was obtained electronically from each participant at the start of the survey.

## Declaration of competing interest

None.
